# An Unrecognised Case of a Scapula Fracture Sustained through an Unusual Indirect Mechanism

**DOI:** 10.1155/2013/848953

**Published:** 2013-09-25

**Authors:** Feiran Wu, Asim Rajpura, Mohammad Shahid, Dilraj Sandher

**Affiliations:** ^1^The Royal Orthopaedic Hospital, Bristol Road South, Birmingham B31 2AP, UK; ^2^University Department of Orthopaedic Surgery, Central Manchester University Hospitals NHS Foundation Trust, Manchester M13 9WL, UK

## Abstract

Scapula fractures following low-velocity injuries are extremely rare but can be a missed associated fracture of other upper limb injuries. We describe the case of a patient who sustained a fracture of the scapula through an unusual and hitherto unreported indirect mechanism. The injury was associated with a radial head fracture and initially missed on presentation. This case highlights the need for increased vigilance when diagnosing injuries with unusual mechanisms.

## 1. Introduction

Fractures of the scapula are rare, comprising 1% of all skeletal injuries and 3%–5% of injuries of the shoulder girdle [1]. As a rule they are sustained as a result of high-velocity trauma, although rarely they can occur due to low impact injuries [2]. We report the case of a patient who sustained a low-velocity indirect fracture of his scapula following a simple mechanical fall, in which the diagnosis was initially missed.

## 2. Report

**Figure 1:**
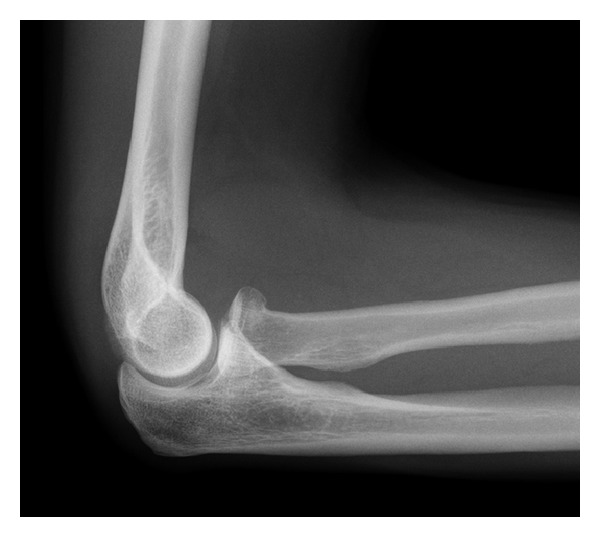
Mason 1 radial head fracture.

A 54-year-old man was referred to clinic with a painful and stiff right shoulder. Six months previously, the patient slipped on ice falling backwards. In order to break his fall, he landed on his right hand with his arm outstretched, abducted, extended, and externally rotated. Following the accident, he attended casualty immediately, where the focus of the examination was on the distracting pain of his elbow but not of the ache in his shoulder. X-rays of the elbow revealed a minimally displaced Mason I right radial head fracture (Figure 1). Radiographs were not taken of the shoulder. He was treated conservatively in a sling for one month before being referred to physiotherapy. Following mobilisation of his arm, he described increasing pain and stiffness in his shoulder.

**Figure 2:**
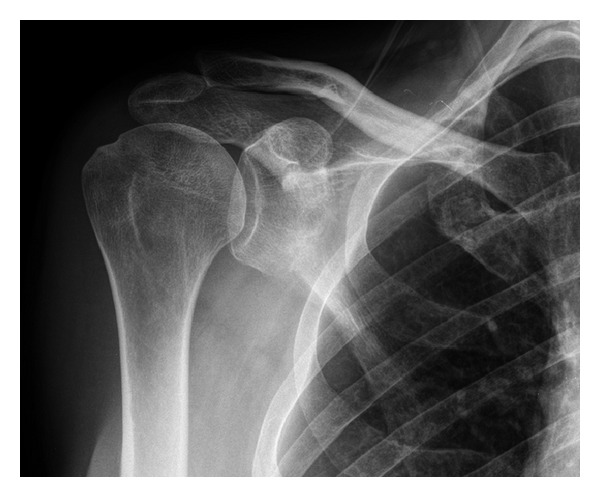
Glenoid neck fracture.

On examination, the patient had a significantly impaired range of motion in all directions. Passive flexion was limited to 90°, abduction to 90°, and lateral rotation to 5°. A diagnosis of frozen shoulder was made. Shoulder X-rays were taken, which revealed a partially united fractured neck of glenoid with minimal displacement. The fracture line extended from the suprascapular notch to the lateral scapula border with impaction at the fracture zone (Figure 2). Since the patient did not recall any other trauma to his upper limb in the past, the cause of this fracture was through an indirect mechanism associated with the radial head fracture secondary to the fall. The patient was treated conservatively with physiotherapy and functional exercises. He made a full recovery of shoulder function and was discharged after 5 months.

## 3. Discussion

Scapula fractures are rare because of both the well-endowed muscular envelope in which the scapula lies and the mobility of the scapula on the thoracic cage. Consequently, glenoid and scapular body fractures typically occur secondary to high-energy trauma, such as vehicular injuries [3]. However, occasionally they can occur as a result of low-velocity injuries. A study by Scavenius over a 10-year period showed 12% of 18 patients with scapula fractures sustained following a fall from the same level [2]. There are also reports of sportsmen sustaining scapula fractures following low-velocity impact [4, 5]. All of these fractures, however, resulted from direct trauma to the lateral or postero superior aspect of the scapula. The present case demonstrates a unique indirect mechanism, in which an axial compressive force was transmitted through the elbow to the glenoid from a lateral and posterior direction when the patient landed on a straight, abducted, and extended arm after falling backwards, fracturing the radial head and glenoid neck. The unusual mechanism may have resulted in a low index of suspicion initially, resulting in the fracture being missed.

Associated injuries occur in 80%–95% of patients who have fractures of the scapula, with the most common being thoracic injuries, lesions to the contralateral extremity, cranial lesions, and spinal lesions [6]. Although fractures of the radial head and neck are common, accounting for 1.7%–5.4% of all adult fractures [7], associated shoulder injuries are rare. Radial head fractures can be classified based on the Mason-Johnston classification [8], with type I fractures treated conservatively with early mobilisation. A study by van Reit and colleagues showed that out of 333 adult patients with radial head fractures, 118 had associated injuries. Of these only 7 (2%) occurred in the shoulder [9]. Interestingly, these were all complications of Mason type I injuries and were rotator cuff strain, acromioclavicular separation, glenohumeral dislocation, and fractures of the clavicle, acromion, and coracoid process. An associated glenoid fracture has never been reported.

This letter highlights a hitherto unreported mechanism for a glenoid fracture. As per advanced trauma life support guidelines, it is imperative to examine neighbouring joints for concomitant injuries. Recognition of this unusual mechanism may help similar such injuries not to be missed. In the current case, an earlier diagnosis of the undisplaced glenoid neck fracture would have resulted in more targeted physiotherapy for the patient which could have avoided the subsequent development of a frozen shoulder and allowed earlier return to normal activity. Although rare, scapula injuries should be in the clinician's mind while examining the associated injuries, especially when the force is directed from a lateral or posterosuperior angle, and radiographs should be sought in all cases where there is any clinical suspicion.
